# Comparison of mid-outcome among bare metal stent, atherectomy with or without drug-coated balloon angioplasty for femoropopliteal arterial occlusion

**DOI:** 10.1038/s41598-023-50511-8

**Published:** 2024-01-02

**Authors:** Lin Yang, Jianjun Quan, Jian Dong, Ningning Ding, Yang Han, Longlong Cong, Yuhao Lin, Jianlin Liu

**Affiliations:** 1 Department of Vascular Surgery, The First Affiliated Hospital of Xi’an Jiaotong University, Xi’an, 710061 China; 2 Department of Vascular Surgery, Hanzhong Central Hospital, Hanzhong, China; 3 Department of Radiology, The First Affiliated Hospital of Xi’an Jiaotong University, Xi’an, China

**Keywords:** Cardiology, Diseases, Medical research, Risk factors, Signs and symptoms

## Abstract

This study evaluated the outcomes of a bare metal stent (BMS), DCB alone, atherectomy plus a drug-coated balloon (AT + DCB) and AT alone for the treatment of femoropopliteal artery occlusion. Four groups were included in this retrospective cohort study: 119 patients underwent the BMS procedure, 89 patients underwent DCB alone, 52 patients underwent AT + DCB, and 61 patients underwent AT alone. Patients were followed-up at 1, 6, 12 and 24 months after the procedure, the clinical outcomes and complications were assessed, and the primary outcomes were primary patency and restenosis. AT + DCB showed a lower bailout stent, and BMS displayed a higher retrograde puncture, flow-limiting dissection and postdilation (*p* < 0.05). For all procedures, the walking distance, ABI and pain score post-procedure were significantly improved compared with the pre-procedure values (*p* < 0.001). The restenosis rate was higher in BMS (21.0%) and AT alone (24.6%) than in DCB (10.1%) alone and AT + DCB (11.5%) (*p* = 0.04); there was no difference in amputation or clinically driven target lesion revascularization among procedures. The primary patency rates were 77.7%, 89.4%, 88.0% and 73.7% in the BMS, DCB alone, AT + DCB and AT alone groups at 24 months, respectively (*p* = 0.03), while the secondary patency and main adverse events (stroke, MI and death) were similar. Proximal concavity, proximal target vessel diameter ≥ 5 mm, runoff number ≥ 2 and DCB use were protective factors for primary patency. Our results suggested that AT + DCB and DCB alone were associated with higher primary patency, and DCB devices (combined with/without AT) should be the preferred choice for FP lesions.

## Introduction

Femoropopliteal (FP) artery stenosis/occlusion is one of the most common lower extremity arterial occlusions, and approximately 10–15% of patients with claudication will develop critical limb-threatening ischemia within 5 years, resulting in a higher risk of amputation and death^1–3^. With the advancement of interventional technology and devices, endovascular procedures have gradually become the first choice for treating FP lesions. Bare metal stent (BMS) and drug-coated balloon (DCB) angioplasty are the most commonly used procedures and can increase the limb salvage rate and patency of the target lesion^4,5^. However, the restenosis rate of BMSs and DCBs is still high due to challenging lesions, such as long-segment occlusion, and BMSs may affect subsequent therapy in the future^6,7^, while long-segment occlusion and significant plaque burden also affect the delivery of anti-proliferative drugs and the long-term outcomes of DCB angioplasty^8–10^.

The atherectomy (AT) procedure can increase the efficiency of DCB and improve the long-term results by removing plaque to obtain more lumen and reduce dissection and bailout stenting. Previous reports have demonstrated that the clinical outcomes of AT combined with DCB are better than those of DCB alone and BMS^11–14^. Further studies have confirmed that the effect of AT followed by DCB angioplasty is better than that of AT followed by ordinary balloon angioplasty. The advantages of AT combined with DCB have been confirmed not only in FP lesions but also in lesions below the knee and severe calcified lesions^15–18^, which could reduce the incidence of flow-limiting dissection and bailout stenting and improve the long-term outcome of DCB angioplasty^19–21^.

However, in the face of long-segment FP occlusion, the best choice of therapy is still one of the main problems faced by vascular physicians. Direct comparative study of BMS, DCB or AT alone and AT plus DCB is the best way to solve this issue. Nevertheless, there is still a lack of literature comparing the clinical outcomes of these procedures. In this retrospective study, we analyzed the procedural and follow-up data of these four procedures in the therapy of complex FP occlusion.

## Methods

### Study design and population

This study is a retrospective cohort study that included patients with chronic total FP occlusion in a university affiliated hospital from January 2019 to June 2021. Patients with symptomatic occlusion lesions in the femoropopliteal artery were eligible, and all patients were diagnosed via computed tomography angiography (CTA) and digital subtraction angiography (DSA). This study was approved by the Ethics Committee of the First Affiliated Hospital of Xi’an Jiaotong University. All patients provided written informed consent, and all information of the participants was collected anonymously. All methods in this study were performed in accordance with relevant guidelines and regulations.

**Figure 1:**
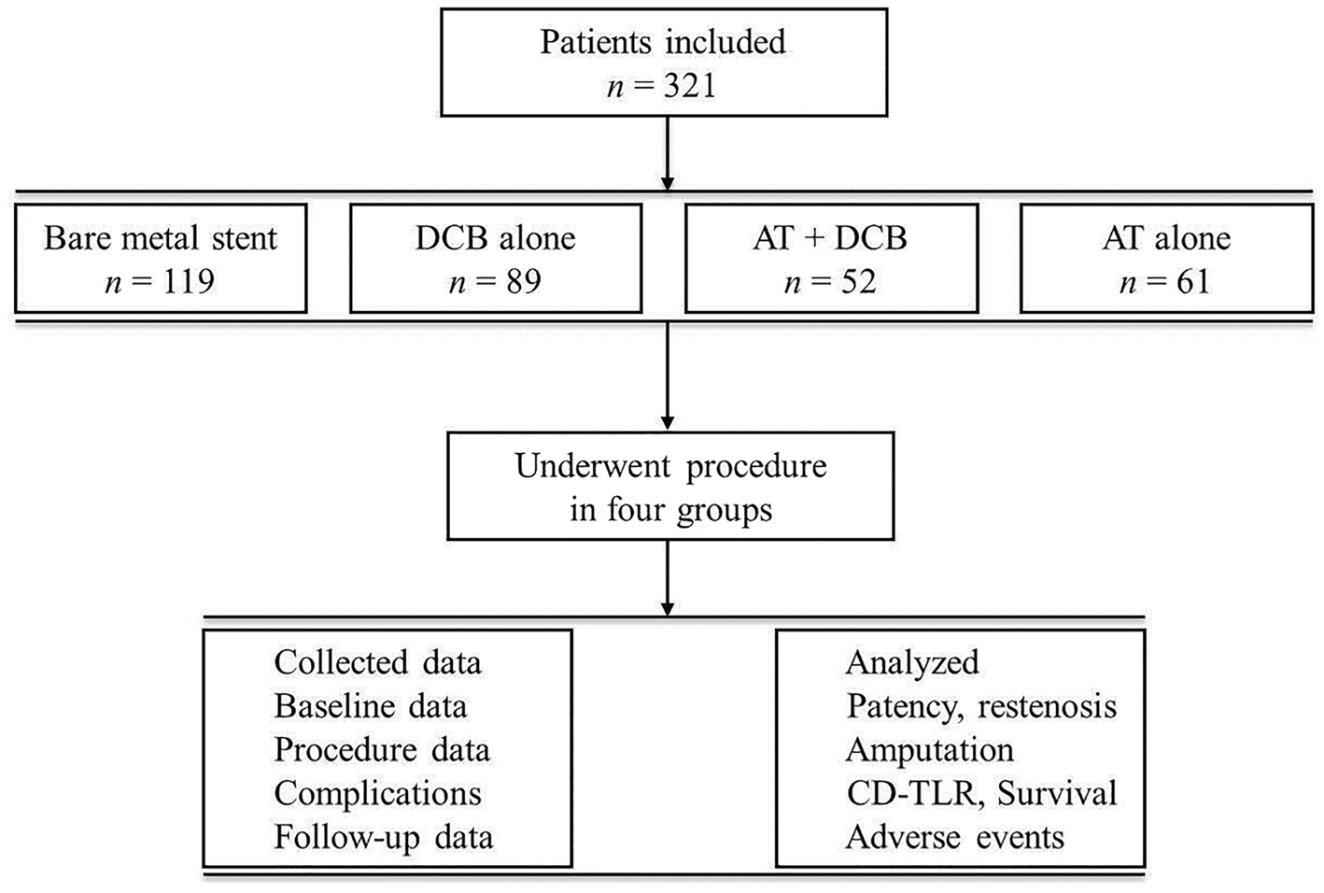
Flowchart of the study procedure. *DCB* drug-coated balloon, *AT* atherectomy, *CD-TLR* clinically driven target lesion revascularization.

A total of 321 patients were included in this study (Fig. 1). All patients were informed in detail about all therapeutic choices and the potential best option for managing the disease, and the final treatment decision was made by the patients and guardians based on their judgment. Finally, 119 patients underwent BMS angioplasty (BMS), 89 patients underwent DCB angioplasty alone (DCB alone), 61 patients were treated with ordinary balloon angioplasty (AT alone), and 52 patients were treated with AT combined with DCB angioplasty (AT + DCB). The clinical data were collected from the medical records, including baseline data, lesion characteristics, complications and outcomes.

The inclusion and exclusion criteria for patient selection are listed as follows.

Inclusion criteria:

1. Patient age: ≥ 18 years;
2. At least one runoff artery below the knee;
3. Rutherford categories 2–6;
4. Length of the target lesion: at least 5 cm;
5. Inflow artery stenosis < 30%;
6. Unilateral lower extremity arterial occlusion;
7. Complete follow-up data.

Exclusion criteria:

1. Arterial embolism of the lower extremity;
2. Inflow artery stenosis ≥ 50% or inflow not successfully treated;
3. Distal outflow occlusions to the target lesion;
4. Previous major amputation of the limb;
5. In-stent reocclusion of the target limb;
6. Severe target limb or systemic infection;
7. Anticipated life expectancy < 1 year;
8. Hypersensitivity or contraindications to paclitaxel, antiplatelet, or anticoagulation therapy;
9. Occlusion involving the common femoral artery and/or beyond the P2 segment of the popliteal artery;
10. Other contraindications to anesthesia or the procedure.

### Endovascular procedures and medicine therapy

All patients underwent endovascular procedures under local anesthesia, and most patients underwent retrograde access of the contralateral femoral artery (or antegrade access when the ipsilateral femoral artery was available) for endovascular procedures. If the guide wire could not pass through the occluded lesion antegradely, retrograde puncture at the distal normal artery segment was used to pass through the lesion, and then the guide wire was confirmed to be located in the distal true lumen via angiography. In the BMS group, ordinary balloon catheters were used to gradually predilate the occlusion lesion of the target vessel, followed by placement of a BMS (laser-engraved bare metal stent, all stents were self-expanding stents). Patients in the DCB alone group were treated with a drug-coated balloon (Orchid, AcoTec, Beijing, China) for final angioplasty after predilation, and bailout stenting (self-expanding stents) was performed if flow-limiting dissection or recoil (> 30%) occurred after DCB angioplasty.

In the AT group, the mechanical atherectomy system was used to obtain a larger lumen, followed by ordinary balloon angioplasty (AT alone) or drug-coated balloon angioplasty (AT + DCB), and bailout stenting was used for cases with flow-limiting dissection or recoil > 30%. Flow-limiting dissection was defined as grade C or above dissection^22^. The final angiography was performed to assess the runoff vessel and embolization after the procedure. Three different AT devices were used in this study: 65 patients (30 with DCB) underwent the laser atherectomy procedure (Turbo‑Elite laser catheter, Spectranetics; Philips Medical Systems, Inc), 21 patients (10 with DCB) underwent the directional atherectomy procedure (Turbo-Hawk, Medtronic, Minneapolis, MN, USA), and 27 patients (12 with DCB) underwent the rotational atherectomy procedure (Straub Medical, Becton Dickenson, NJ, USA).

All patients received dual antiplatelet therapy consisting of aspirin (100 mg/d) and clopidogrel (75 mg/d) for at least 3 days before the procedure and then continued to take dual antiplatelet medical therapy (aspirin 100 mg/d and clopidogrel 75 mg/d) for 6 months after the procedure. Meanwhile, atorvastatin (20 mg/d) was given for lipid-lowering therapy, and beraprost sodium was given for adjuvant medicine therapy (120 µg/d), followed by long-term oral single antiplatelet drug and statin therapy.

### Assessment and definition

All patients were followed-up at 30 days and 6, 12 and 24 months after the procedure. Follow-up data included clinical symptoms, the Rutherford classification, ankle brachial index (ABI) values and ultrasound and/or CTA results. The morphology of proximal lesions was defined as concave proximal and convex proximal. The cutoff time for safety and clinical endpoint analysis was 24 months. The primary outcomes were the patency of target vessels at 24 months after the procedure. Primary patency was defined as a target lesion without obvious restenosis (< 50%) or clinically driven target lesion revascularization (CD-TLR); secondary patency was defined as patency maintained after secondary endovascular therapy in patients with reocclusion after the procedure. The secondary outcomes were amputation rate, all-cause mortality and CD-TLR, and CD-TLR was defined as any repeated endovascular therapy or bypass surgery associated with Rutherford grade deterioration and/or an increase in the original wound size and/or new wound appearance^22–24^. Significant restenosis was defined as > 50% based on angiography- or ultrasound examination-derived velocity parameters (peak systolic velocity ratio ≥ 2.4). The major adverse events (MAEs) included myocardial infarction (MI), stroke and all-cause death. Limb pain was assessed by the visual analog scale (VAS), with a score ranging from 0 to 10, and a higher value indicated more severe pain^25^. The calcification degree was assessed by using the proposed peripheral arterial calcium-scoring system (PACSS). This system included grades 0 to 4; grades 0 and 1 were defined as non/mild calcification, grade 2 was defined as medium calcification, and grades 3–4 were defined as severe calcification^26^.

### Statistical analysis

All data were collected in an Excel file (Version 2013, Microsoft, Redmond, Washington) and analyzed using SPSS v. 22.0 (SPSS, Chicago, IL, USA) software. Categorical variables are presented as numbers (percentages) and were compared using the Chi-square test or Fisher’s exact test. Continuous variables are presented as the mean and standard deviation (SD). The normal distribution was tested by using the Shapiro‒Wilk test and then compared by using one-way analysis of variance and Student–Newman‒Keuls test. The primary patency, secondary patency and overall survival rate of patients were analyzed using the Kaplan‒Meier method and were compared by the log-rank test. To confirm risk factors for restenosis, univariate and multivariate Cox hazard regression analyses were performed with all baseline and procedural variables. The variate with *p* < 0.20 was used as the covariate after univariate analysis, multivariate analysis was performed, and the hazard ratio (HR) and 95% confidence interval (CI) of risk factors were calculated. *p* values < 0.05 were considered statistically significant.

## Results

### Demographic characteristics and baseline data

This study included a total of 321 limbs of 321 patients with FP occlusion (Fig. 1); 119 patients received the BMS procedure, 89 patients received DCB angioplasty alone, 52 patients underwent AT combined with DCB angioplasty (AT + DCB), and 61 patients underwent AT angioplasty alone. There were no significant differences in demographic and baseline characteristics among these four procedures (Table 1). No differences in Rutherford category, pre-ABI, claudication distance, VAS pain score or medicine therapy were confirmed among the four procedures (*p* > 0.05).

**Table 1:** The demographic and baseline characteristics of the four procedures.

	BMS (n = 119)	DCB (n = 89)	AT + DCB (n = 52)	AT (n = 61)	*p* value*
Gender (M)	100 (84.0)	66 (74.2)	47 (90.4)	46 (75.4)	0.06
Age (year)	68.74 ± 10.74	68.11 ± 7.82	68.13 ± 7.75	68.30 ± 11.31	0.96
BMI (kg/m^2^)	22.91 ± 2.97	23.41 ± 2.94	23.35 ± 2.92	22.80 ± 3.07	0.49
Lesion side (L)	68 (57.1)	44 (49.4)	28 (53.8)	27 (43.5)	0.39
Rutherford category					0.40
Level 3	56 (47.1)	43 (48.3)	33 (63.5)	29 (47.5)	
Level 4	45 (37.8)	28 (31.5)	11 (21.1)	19 (31.2)	
Level 5	10 (8.4)	13 (14.6)	4 (7.7)	6 (9.8)	
Level 6	8 (6.7)	5 (5.6)	4 (7.7)	7 (11.5)	
Claudication distance	184.79 ± 161.77	170.62 ± 141.50	185.58 ± 170.07	167.87 ± 144.04	0.85
Pre-ABI	0.28 ± 0.18	0.28 ± 0.17	0.30 ± 0.18	0.30 ± 0.17	0.81
Pre-VAS	5.31 ± 1.53	5.52 ± 1.62	5.52 ± 1.29	5.72 ± 1.36	0.36
Smoking	71 (59.7)	48 (53.9)	38 (73.1)	35 (57.4)	0.16
Drinking	13 (10.9)	13 (14.6)	11 (21.1)	9 (14.8)	0.38
CAD	40 (33.6)	28 (31.5)	15 (28.8)	15 (24.6)	0.64
Stroke	34 (28.6)	27 (30.3)	12 (23.1)	11 (18.0)	0.32
PAD history	23 (19.3)	13 (14.6)	11 (21.1)	12 (19.7)	0.71
Hypertension	77 (64.7)	56 (62.9)	30 (57.7)	42 (68.9)	0.66
Type 2 diabetes	55 (46.2)	45 (50.6)	27 (51.9)	24 (39.3)	0.49
COPD	2 (1.7)	2 (2.2)	3 (5.8)	1 (1.6)	0.42
Hyperlipidemia	56 (47.1)	37 (41.6)	27 (51.9)	22 (36.1)	0.32
H-HCY	21 (17.7)	17 (19.1)	8 (15.4)	18 (29.5)	0.20
Tumor	2 (1.7)	2 (2.2)	1 (1.9)	2 (3.3)	0.91
Other	3 (2.5)	2 (2.2)	2 (3.8)	3 (4.9)	0.78
Aspirin	111 (93.2)	83 (93.3)	46 (88.5)	59 (96.7)	0.38
Clopidogrel	113 (95.0)	77 (86.5)	49 (94.2)	57 (93.4)	0.13
Cilostazol	2 (1.7)	6 (6.7)	1 (1.9)	1 (1.6)	0.15
Sagrelate	4 (3.4)	7 (7.9)	3 (5.8)	1 (1.6)	0.27
Rivaroxaban	12 (10.1)	9 (10.1)	5 (9.6)	4 (6.6)	0.87
Beraprost sodium	92 (77.3)	70 (78.7)	39 (75.0)	53 (86.9)	0.39
Atorvastatin	117 (98.3)	88 (98.9)	50 (96.2)	58 (95.1)	0.41

*BMS* bare metal stent, *DCB* drug-coated balloon, *AT* atherectomy, *M* male, *BMI* body mass index, *kg* kilogram, *L* left, *ABI* ankle brachial index, *VAS* visual analog scale, *CAD* coronary atherosclerotic disease, *PAD* peripheral arterial diseases, *COPD* chronic obstructive pulmonary disease, *H-HCY* hyperhomocysteinemia. *p value, comparison of four procedures.

### Lesion and procedural characteristics

The occluded lesion and procedural characteristics are listed in Table 2. In this case cohort study, all lesions were occlusions, and the main manifestations were TASC C/D lesions. The proximal and distal diameters of the target vessels were similar among the four procedures. There was no difference in the morphological type of the proximal plaque cap, runoff number of BTK, predilation, bailout stent, distal embolization or closure device (*p* > 0.05), while the rate of bailout stent in the AT + DCB procedure was significantly less than that in the DCB and AT alone procedures (*p* < 0.05). However, a higher retrograde puncture rate, postdilation, flow-limiting dissection and stent number were confirmed in the BMS procedure than in the other three procedures (*p* < 0.01), and the procedure time in the DCB-alone procedure was higher than that in the other three procedures (*p* < 0.05).

**Table 2:** Lesion and procedural characteristics of the four procedures.

	BMS (n = 119)	DCB (n = 89)	AT + DCB (n = 52)	AT (n = 61)	*p* value*
TASC					0.24
A	2 (1.7)	4 (4.4)	0 (0)	0 (0)	
B	26 (21.8)	20 (22.5)	9 (17.3)	15 (24.6)	
C	19 (16.0)	20 (22.5)	9 (17.3)	6 (9.8)	
D	72 (60.5)	45 (50.6)	34 (65.4)	40 (65.6)	
PACSS					0.96
Non/mild	39 (32.8)	26 (29.2)	15 (28.9)	18 (29.5)	
Medium	43 (36.1)	35 (39.3)	19 (36.5)	21 (34.4)	
Severe	37 (31.1)	28 (31.5)	18 (34.6)	22 (36.1)	
Iliac involved	17 (14.3)	6 (6.7)	5 (9.6)	4 (6.6)	0.23
BTK involved	32 (26.9)	34 (38.2)	16 (30.8)	18 (29.5)	0.37
Proximal diameter (mm)	5.35 ± 0.93	5.24 ± 0.90	4.83 ± 0.78	4.93 ± 0.74	0.52
Distal diameter (mm)	4.94 ± 0.90	4.88 ± 0.73	4.44 ± 0.62	4.47 ± 0.73	0.39
Length of occlusion (cm)	20.20 ± 10.65	18.28 ± 9.25	20.66 ± 9.12	21.81 ± 10.78	0.19
Runoff number					0.99
< 2	73 (61.3)	55 (61.8)	33 (63.5)	38 (62.3)	
≥ 2	46 (38.7)	34 (38.2)	19 (36.5)	23 (37.7)	
Proximal morphology					0.23
Concave	77 (64.7)	66 (74.2)	38 (70.8)	37 (60.7)	
Convex	42 (35.3)	23 (25.8)	14 (29.2)	24 (39.3)	
Retrograde puncture	25 (21.0)	8 (9.0)	2 (3.8)	5 (8.2)	0.004
Predilation	118 (99.2)	89 (100)	52 (100)	60 (98.4)	0.57
Postdilation	43 (36.1)	5 (5.6)	2 (3.8)	5 (8.2)	< 0.001
Flow-limiting dissection	32 (26.8)	11 (12.3)	2 (3.8)	7 (11.4)	< 0.001
Bailout stent	0 (0)	14 (15.7)	2 (3.8)	12 (19.7)	0.18
Closure device	100 (84.0)	68 (76.4)	43 (84.3)	50 (82.0)	0.51
Stent number	1.8 ± 0.8	0.2 + 0.5	0.1 ± 0.2	0.7 ± 0.9	< 0.001
Procedure time (h)	3.14 ± 0.60	2.87 ± 0.54	3.02 ± 0.49	3.06 ± 0.51	0.01

*BMS* bare metal stent, *DCB* drug-coated balloon, *AT* atherectomy, *TASC* trans-Atlantic intersociety consensus, *PACSS* proposed peripheral arterial calcium-scoring system, *mm* millimeter, *cm* centimeter, *h* hour. *p value, comparison of four procedures.

### Short-term and mid-term outcomes and complications

All patients completed the endovascular procedure, and the technical success rate was 100%. Perforation and distal embolization occurred in the AT and DCB procedures, but no differences were confirmed (Table 3). Perforation was treated via stent angioplasty, and distal embolization was treated via a thrombus aspiration catheter. There were no significant differences in the access complications or main adverse events (stroke, MI, death and deep vein thrombosis) at 1 month (*p* > 0.05). The postwalking distance, post-ABI and post-VAS score were similar in the four procedures.

**Table 3:** Short-term and mid-term outcomes of the procedures.

	BMS (n = 119)	DCB (n = 89)	AT + DCB (n = 52)	AT (n = 61)	*p* value*
Technique success	119 (100)	89 (100)	52 (100)	61 (100)	Null
Hematoma	3 (2.5)	1 (1.1)	1 (1.9)	2 (3.2)	0.83
Pseudoaneurysm	1 (0.8)	1 (1.1)	0 (0)	1 (1.6)	0.83
Bleeding	1 (0.8)	1 (1.1)	1 (1.9)	0 (0)	0.76
Perforation	0 (0)	0 (0)	1 (1.9)	1 (1.6)	0.29
Distal embolization	0 (0)	1 (1.1)	1 (1.9)	2 (3.2)	0.29
Outcome @ 1 mon					
Death	0 (0)	0 (0)	0 (0)	0 (0)	Null
MI	0 (0)	0 (0)	0 (0)	0 (0)	Null
Stroke	0 (0)	0 (0)	0 (0)	0 (0)	Null
DVT	0 (0)	0 (0)	0 (0)	0 (0)	Null
Postwalking distance (m)	1105.4 ± 691.1	1345.3 ± 868.2	1132.0 ± 813.0	1337.7 ± 975.2	0.36
Post-ABI	0.97 ± 0.17	0.98 ± 0.16	1.00 ± 0.15	1.02 ± 0.16	0.42
Post-VAS	0.93 ± 1.09	0.66 ± 0.87	1.00 ± 0.97	1.02 ± 1.06	0.11
Outcome @Follow-up					
Follow-up time (mon)	23.40 ± 6.80	23.87 ± 7.48	24.21 ± 5.85	25.00 ± 6.11	0.49
Restenosis	25 (21.0)	9 (10.1)	6 (11.5)	15 (24.6)	0.04
CD-TLR	20 (16.8)	8 (9.0)	5 (9.6)	13 (21.3)	0.09
Amputation	3 (2.5)	3 (3.4)	1 (1.9)	3 (4.9)	0.78
Cumulative death	7 (5.8)	4 (4.5)	2 (3.8)	4 (6.5)	0.89
Cumulative stroke	1 (0.8)	2 (2.2)	1 (1.9)	4 (6.5)	0.13
Cumulative MI	6 (5.0)	1 (1.1)	1 (1.9)	4 (6.5)	0.25

*BMS* bare metal stent, *DCB* drug-coated balloon, *AT* atherectomy, *MI* myocardial infarction, *DVT* deep vein thrombosis, *ABI* ankle brachial index, *VAS* visual analog scale, *mon* months, *CD-TLR* clinically driven target lesion revascularization. *p value, comparison of four procedures.

The walking distance and ABI after the procedure were significantly higher than those before the procedure among the four procedures (*p* < 0.001), and the VAS pain score was significantly decreased after the procedure compared with before the procedure (*p* < 0.001). The restenosis rate was higher in the BMS (21.0%) and AT alone (24.6%) groups than in the DCB (10.1%) alone and AT + DCB (11.5%) groups (*p* = 0.04); there was no difference in the CD-TLR rate among the four procedures; however, a higher CD-TLR tendency was confirmed in the BMS (16.8%) and AT alone (21.3%) groups (*p* = 0.09). No difference was confirmed in the amputation rate among the four procedures (*p* > 0.05).

**Figure 2:**
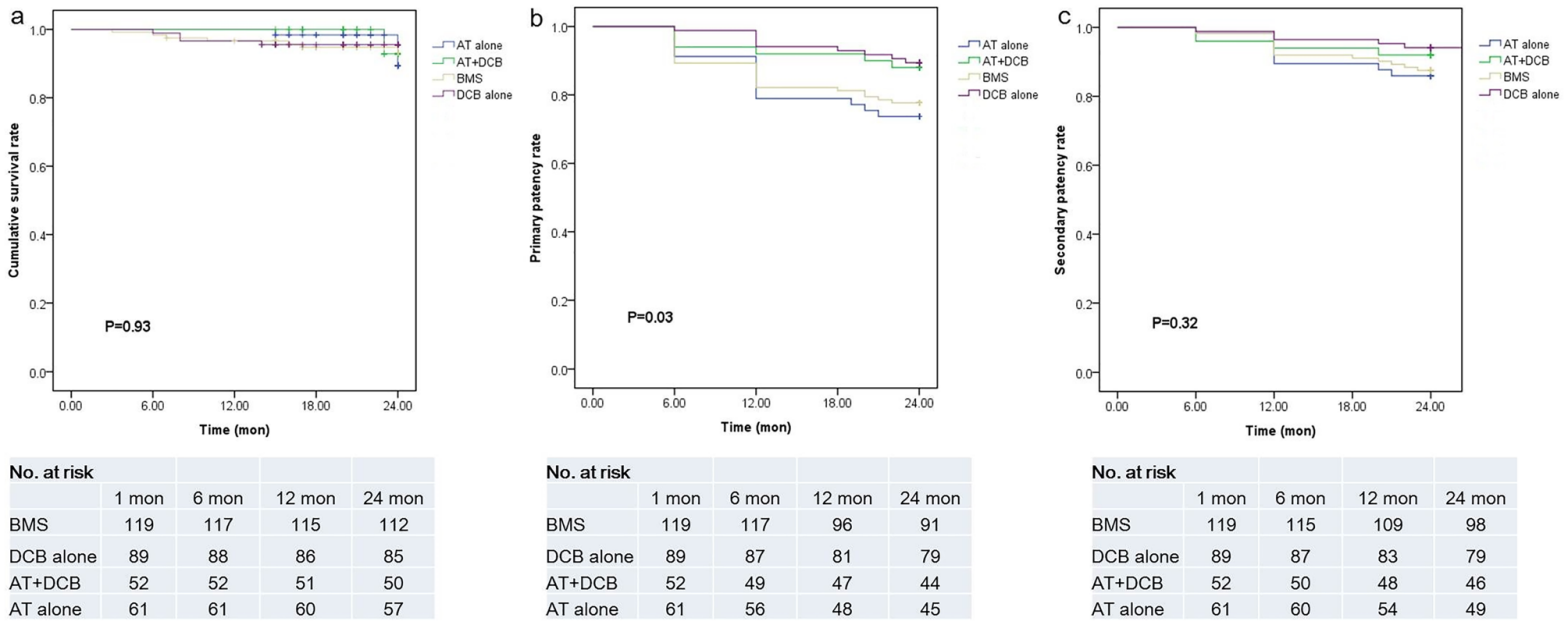
Kaplan‒Meier survival analysis for cumulative survival (**a**), primary (**b**) and secondary (**c**) patency rates of the four procedures. The number at risk represents the number of evaluable participants at the beginning of each follow-up interval. *BMS* bare metal stent, *DCB* drug-coated balloon, *AT* + *DCB* atherectomy + drug-coated balloon, *AT* atherectomy, *mon* month, *No*. number.

The cumulative stroke, MI and death rates were similar among the four procedures, and no difference was confirmed between the procedures (p > 0.05). The survival rates were 94.1%, 95.5%, 96.1% and 93.4% in the BMS, DCB alone, AT + DCB and AT alone procedures at 24 months, respectively (Fig. 2a, p > 0.05). The primary patency of the target lesion was 77.7%, 89.4%, 88.0% and 73.7% in the BMS, DCB alone, AT + DCB and AT alone groups at 24 months, respectively. The primary patency in the DCB alone and AT + DCB groups was higher than that in the BMS and AT alone groups (Fig. 2b, p = 0.03), while the secondary patency rates were 87.5%, 92.9%, 92.0% and 85.9% in the BMS, DCB alone, AT + DCB and AT alone groups at 24 months, respectively (Fig. 2c, p > 0.05).

### Univariate and multivariate analyses for predictors of restenosis

Univariate regression analysis was used to determine the potential risk factors related to restenosis (Table 4), and multivariate regression analysis was used to confirm the independent risk factors for restenosis. A prewalking distance < 50 m (HR: 1.95; 95% CI: 1.03–3.67, *p* = 0.04) was an independent risk factor for restenosis. Proximal concavity (HR: 0.49; 95% CI: 0.27–0.89, *p* = 0.02), proximal target vessel diameter ≥ 5 mm (HR: 0.32; 95% CI: 0.12–0.85, *p* = 0.02), runoff number ≥ 2 (HR: 0.45; 95% CI: 0.22–0.91, *p* = 0.02) and DCB use (HR: 0.24, 95% CI: 0.11–0.57, *p* = 0.001) were potential protective factors for restenosis.

**Table 4:** Univariate and multivariate regression analysis for predictors of restenosis.

	Univariate analysis			Multivariate analysis		
	HR	95% CI	*p* value	HR	95% CI	*p* value
Post-ABI < 0.85	2.23	1.16–4.44	0.02			
Predistance < 50 m	1.80	0.96–3.38	0.06	1.95	1.03–3.67	0.04
PAD history	2.14	1.09–4.2	0.02			
Type 2 diabetes	1.86	1.03–3.35	0.04			
Proximal concave	0.68	0.38–1.25	0.20	0.49	0.27–0.89	0.02
Proximal dia. ≥ 5 mm	0.43	0.23–0.80	0.007	0.32	0.12–0.85	0.02
Distal dia. ≥ 4.5 mm	0.41	0.23–0.76	0.004			
TASC C/D	1.63	0.88–3.01	0.11			
Runoff number ≥ 2	0.86	0.47–1.58	0.13	0.45	0.22–0.91	0.02
Severe calcification	2.14	1.19–3.85	0.01			
DCB used	0.39	0.21–0.74	0.003	0.24	0.11–0.57	0.001
Postdistance	2.57	1.40–4.72	0.002			

*BMS* bare metal stent, *DCB* drug-coated balloon, *AT* atherectomy, *HR* hazard ratio, *95% CI* 95% confidence interval, *ABI* ankle brachial index, *m* meter, *PAD* peripheral arterial diseases, *mm* millimeter, *TASC* trans-Atlantic intersociety consensus, *DCB* drug-coated balloon, *VAS* visual analog scale.

## Discussion

Long-segmental occlusion of FP lesions is still a challenging problem for PAD therapy, in which BMS plays a major role. AT technology using different plaque removal systems is more conducive to obtaining the lumen and reducing the incidence of flow-limiting dissection, but the effectiveness of the application of AT alone is still controversial^14,27,28^, and some studies have proven that the clinical outcome of AT combined with the DCB procedure is better^11,12^. However, there is still a lack of head-to-head comparisons of BMS, DCB alone, AT + DCB, and AT alone procedures to determine the best therapy modality for FP lesions. Our study showed that the survival rate, CD-TLR and secondary patency of the four procedures were not significantly different, but AT combined with DCB and DCB alone revealed a higher primary patency rate. The walking distance, ABI and post-VAS pain score of the four procedures were significantly improved compared with those before the procedure, and there was no difference in complications, amputation rate or major adverse events for the four procedures. These data confirmed that these techniques remain effective therapeutic methods for FP lesions, but the BMS procedure has a higher incidence of flow-limiting dissection, postdilatation and restenosis.

Compared with traditional angioplasty procedures, DCB angioplasty improves the long-term patency rate and limb salvage rate of patients, but complex lesions such as long-segment occlusion, restenosis and severe calcification may affect the drug delivery efficiency of DCB balloons, thus affecting the outcome of the procedure^9,10,29,30^. However, the AT procedure could improve the effect of vessel preparation and is more conducive to the use of DCBs. Further studies have shown that AT devices combined with DCBs show higher patency^21,31,32^. Our study demonstrated that the AT procedure followed by DCB angioplasty has a higher primary patency and lower restenosis rate for FP occlusion. The ordinary BMS procedure indicated a higher incidence of flow-limiting dissection when compared with DCB alone, and AT combined with the DCB procedure showed lower rates of flow-limiting dissection and bailout stenting. Although some previous reports have indicated that AT combined with the DCB procedure may improve primary patency^11,12,15^, our study demonstrated that there was no difference between AT combined with DCB and DCB alone in terms of primary patency.

The AT device can reduce the plaque/thrombus load of occlusive lesions, thereby obtaining larger lumens, which is more conducive to the delivery and utilization of DCB drugs. Previous studies confirmed that the primary patency of AT combined with DCB is significantly higher than that of DCB alone and BMS^11,21^. More importantly, this procedure can preserve potential opportunities for future therapy. The long-term patency of the AT procedure combined with ordinary balloon/stent angioplasty does not show a corresponding advantage. Some studies have even shown that the primary patency of AT combined with ordinary balloon angioplasty is not better than that of balloon angioplasty alone^14^. Another study suggests that the long-term outcome of AT combined with DCB angioplasty is significantly better than that of AT combined with ordinary balloon angioplasty or DCB angioplasty alone^33^. Our data revealed that the primary patency of AT alone followed by balloon or bailout stenting is lower than that of AT combined with DCB. Further studies have confirmed that the bailout stenting of AT combined with DCB is also significantly lower than that of AT alone and DCB alone^14,33^. Therefore, DCB (combined with/without AT) should be the first choice for long-segment FP occlusion in clinical practice.

Restenosis is the main factor affecting the long-term patency of FP lesions. Several studies have found that severe calcification, long-segment occlusion, in-stent restenosis, TASC C/D lesions, runoff number and other factors are closely related to restenosis^29,34,35^. In our study, we determined the potential risk factors by regression analysis. A prewalking distance < 50 m was an independent risk factor for restenosis, while proximal concave lesion, proximal artery diameter ≥ 5 mm and runoff number ≥ 2 were protective factors. These data are consistent with most of the conclusions from previous studies. Our study found that the morphology of the proximal concave occlusion was associated with a lower retrograde puncture rate and incidence of restenosis. These data suggest that the morphology of the occlusion cap may have a potential impact on the technical success and clinical outcomes of angioplasty, while larger proximal diameters and more runoff vessels indicate a lower incidence of restenosis. Furthermore, our data confirmed that the use of DCB balloons is the key factor in reducing the incidence of restenosis and maintaining long-term patency.

### Limitations

Although this study conducted a head-to-head comparison of the four procedures of BMS, DCB alone, AT + DCB and AT alone on FP occlusion lesions, there are also obvious limitations in this study. First, this study is not a prospective, randomized controlled trial, and its results may be affected by potential confounding factors. Second, this study is not a multicenter data analysis, and the selection bias of patients and devices used may affect the generalizability of the conclusions. Third, our research did not include all the devices being used; thus, our findings need further verification. Our conclusions still need to be confirmed by multicenter, prospective, large-sample RCT studies in the future.

## Conclusions

Our data suggest that the DCB procedure (combined with/without AT) shows higher primary patency and that the AT combined with DCB procedure has a lower incidence of flow-limiting dissection and bailout stenting; thus, DCB devices (combined with/without AT) should be the first therapeutic choice for patients with FP lesions. We believe that this study can provide some evidence for the debate on the best procedure for FP occlusion.

## Data Availability

The data from this study are available from the corresponding author upon reasonable request.
